# Seroepidemiological study of Japanese encephalitis virus in Chiang Mai: Immunity and susceptibility 28 years after introduction of a vaccination programme

**DOI:** 10.1371/journal.pntd.0010674

**Published:** 2022-08-01

**Authors:** Tavitiya Sudjaritruk, Quanhathai Kaewpoowat, Chanidapa Prasarakee, Saowalak Sarachai, Anne-Frieda Taurel, Natthanidnan Sricharoen, Phatraporn Assawawongprom, Jutamad Saheng, Rebecca Harris, Joshua Nealon, Sutee Yoksan

**Affiliations:** 1 Department of Pediatrics, Faculty of Medicine, Chiang Mai University, Chiang Mai, Thailand; 2 Clinical and Molecular Epidemiology of Emerging and Re-emerging Infectious Diseases Research Cluster, Faculty of Medicine, Chiang Mai University, Chiang Mai, Thailand; 3 Research Institute for Health Sciences, Chiang Mai University, Chiang Mai, Thailand; 4 Department of Internal Medicine, Faculty of Medicine, Chiang Mai University, Chiang Mai, Thailand; 5 Vaccine Epidemiology and Modeling Department, Sanofi, Singapore; 6 Medical Department, Sanofi, Bangkok, Thailand; 7 Center for Vaccine Development, Institute of Molecular Biosciences, Mahidol University, Bangkok, Thailand; 8 Chulabhorn Research Institute, Bangkok, Thailand; University of Pittsburgh, UNITED STATES

## Abstract

**Background:**

Thailand has introduced a nationwide vaccination against Japanese encephalitis virus (JEV) into National Immunization Programme since the 1990’s. To improve the understanding of immunity and susceptibility of the population after 28 years of a vaccination programme, we conducted a JEV seroepidemiological study in a JEV-endemic area of Thailand.

**Methods:**

An age-stratified, population-based, seroepidemiological study was conducted in Chiang Mai, Thailand–a northern Thai province where is an endemic area of Japanese encephalitis. Nine districts were chosen based on administrative definition: rural (*n* = 3); urban (*n* = 3); and peri-urban (*n* = 3). Within each district, eligible participants were randomly selected from 3 age groups: adolescents (10–20 years); adults (21–50 years); and older adults/elderly (≥51 years) by computer randomization. Plaque reduction neutralization tests (PRNT_50_ and PRNT_90_) were performed to measure neutralizing antibodies to JEV. To account for the cross-reactivity of JEV and other flaviviruses, JEV seroprotection was defined according to age, previous history of JEV vaccination, and PRNT_50_/PRNT_90_ levels of study participants.

**Results:**

Overall, 279 adolescents, 297 adults, and 297 older adults/elderly were enrolled from nine districts. Age-stratified, protocol-defined, cluster-adjusted JEV seroprotection rates were 61% (95% CI: 48–73%), 43% (95% CI: 31–57%), and 52% (95% CI: 37–67%) for adolescents, adults, and older adults/elderly, respectively. Living in peri-urban districts, having a history of prior dengue virus infection, and previously receiving mouse brain-derived JEV vaccine were significantly associated with seroprotection to JEV in adolescents. Older age and male sex were associated with seroprotection for adults; and only male sex was the associated factor for older adults/elderly (*P* <0.05).

**Conclusions:**

Approximately half of population living in a JEV-endemic area demonstrated seroprotection to JEV. Ongoing nationwide surveillance on JEV seropepidemiology is an important strategy to understand the evolving population-level immunity to JEV, and to help formulating the appropriate recommendations on JE immunization.

## Author summary

Japanese encephalitis virus (JEV) is a mosquito-borne virus which is the leading cause of encephalitis, namely Japanese encephalitis (JE), in Southeast Asia and the Western Pacific region. To reduce the burden of disease, Thailand has introduced a nationwide vaccination against JEV into National Immunization Program (NIP) since the 1990’s. Although JE is endemic in Thailand, there have been a limited number of JEV seroepidemiological studies in Thai populations because of the lack highly specific serological assays which account for the cross-reactivity between JEV and other members of the flavivirus family. Thus, we conducted this study which primarily aimed to improve the understanding of immunity and susceptibility of the population living Chiang Mai, a northern Thai province and a JEV-endemic area, after 28 years of a vaccination program to guide the implementation of JE prevention and control measures. Our results suggest that, despite the commendable vaccination effort over the past 3 decades, approximately half of general population (39% of adolescents, 57% of adults, and 48% of older adults/elderly) remains susceptible to JEV infection. Ongoing nationwide surveillance on JEV seropepidemiology is an important strategy to understand the evolving population-level immunity to JEV, and to help formulating the appropriate recommendations on JE immunization.

## Introduction

Japanese encephalitis virus (JEV), a mosquito-borne virus belonging to the genus *Flavivirus* of the family *Flaviviridae*, is the leading cause of encephalitis in Southeast Asia and the Western Pacific region [1,2]. JEV is maintained in a zoonotic cycle between *Culex* mosquitoes, predominantly *Culex tritaeniorhynchus*, and vertebrate hosts, primarily wading birds and pigs which act as natural reservoirs and amplifying hosts. Humans are incidental dead-end hosts who are at risk of infection when living in close proximity with the indicated vertebrate hosts [3,4]. JEV transmission is associated with ecological risk factors which are mainly found in rural and peri-urban agricultural areas [3], but transmission can also occur in urban centers in some Asian countries [5–7].

Thailand is an endemic area for JE, reporting 1,500 to 2,500 cases annually in the 1970’s and 1980’s [8,9]. To reduce the burden of disease, the Thailand Ministry of Public Health (MOPH) introduced stepwise vaccination against JEV in the 1990’s, beginning with two primary doses of mouse brain-derived JEV vaccine (MBDV; JE-VAX, Thai Governmental Pharmaceutical Organization [TGPO], Beijing strain) in children aged 18–24 months [10,11]. However, with suboptimal seroconversion rates, a third dose was added to the routine immunization schedule for children aged 30 months in 2000 [12]. Vaccination coverage for 3 doses of MBDV was 62% among children aged 3–4 years in 2003, which increased to 89% in 2008 [13]. Notably, the vaccine effectiveness of 3-dose MBDV within 3 years of the final dose was 95% (95% confidence interval [95% CI]: 80–99%) among children aged ≥18 months [13]. In 2016, a full 3-dose series of MBDV was replaced by a 2-dose series of live-attenuated JEV vaccine (LAJEV; CD.JEVAX, Chengdu Institute of Biological Products, SA 14-14-2 strain), of which the vaccine effectiveness in China was estimated at 98% (95% CI: 86–100%) among children <15 years [14]. According to the 2018 childhood National Immunization Coverage Survey, the vaccination coverage for 2 doses of LAJEV was 95% [15]. After the implementation of a nationwide JE vaccination program in Thailand, the annual incidence of JE has greatly diminished to less than 500 cases since the late 1990’s [16].

Although JE is endemic in Thailand, there have been a limited number of JEV seroepidemiological studies in Thai populations [17–19]. These studies are challenging in areas where multiple flaviviruses co-circulate, and high coverage of JE vaccination is achieved, because we lack highly specific serological assays which account for the cross-reactivity between JEV and other members of the flavivirus family [20–22]. Plaque reduction neutralization test (PRNT) is a gold standard in measuring protective antibody against JEV, but there is currently no consensus on the optimum plaque reduction (%) among clinical laboratories [23]. Generally, PRNT_50_ is used to define seroprotection of JEV; however, PRNT_90_ may be preferable for seroepidemiological studies in populations who have already been vaccinated against JEV, and/or have a high chance of other flavivirus exposure [24,25].

Between 2010 and 2017, Chiang Mai, a northern Thai province, was ranked in the top 10 provinces with the highest incidence rates of JE and unspecified encephalitis four times. The average annual incidence of disease was about 2 cases per 100,000 population [16]. The significant burden of JE in Chiang Mai indicates the need for seroepidemiological studies to guide the implementation of JE prevention and control measures. This study primarily aimed to estimate an age-stratified proportion of the population seroprotected against JEV, based on immunity from previous vaccination or prior exposure to the disease. We then aimed to identify socio-demographic factors associated with JEV seroprotection among these populations.

## Methods

### Ethics statement

This study was approved by the Research Ethics Committee of the Faculty of Medicine, Chiang Mai University (COA no. 397/2018). All participants and their caregivers (if participants aged <18 years) provided written informed consent and assent, as appropriate, prior to study enrollment.

### Study design

**Fig 1:**
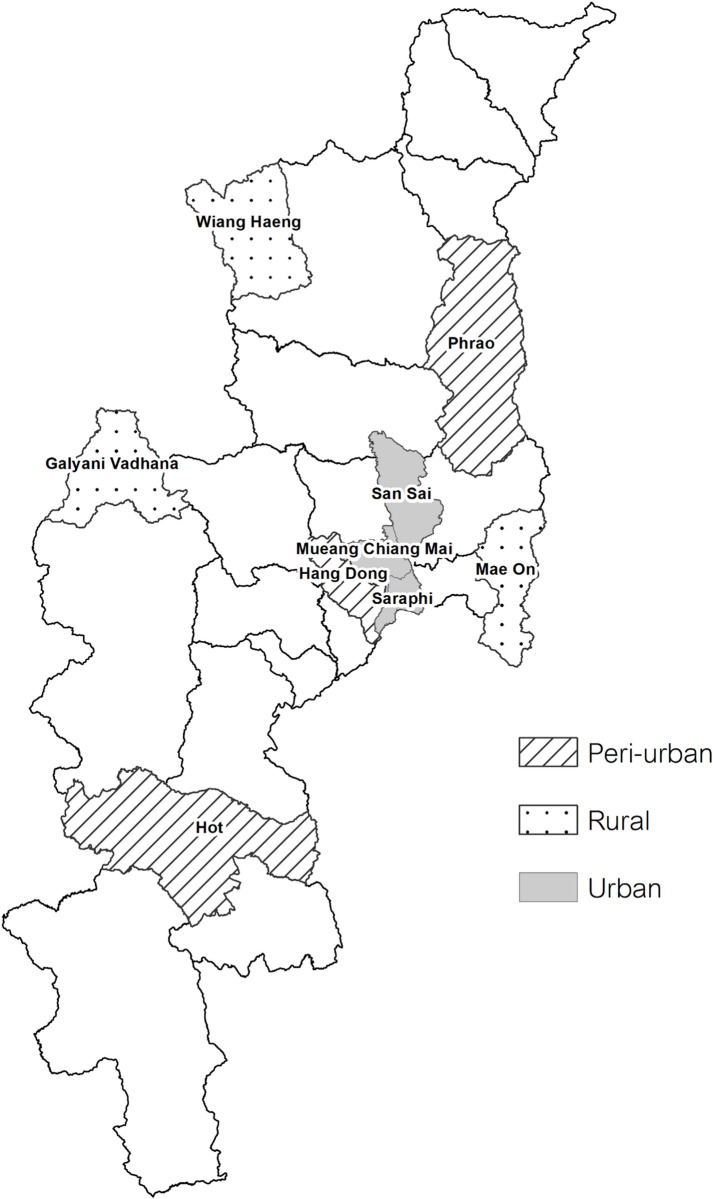
Geographic location of the nine research clusters in Chiang Mai, Thailand. Nine research clusters included (1) rural district (*n* = 3): Wiang Haeng, Mae On, and Galyani Vadhana; (2) urban district (*n* = 3): Mueang, Saraphi, and San Sai; and (3) peri-urban district (*n* = 3): Hang Dong, Hot, and Phrao, according to the 2010 population and housing census of Thailand. Note: The base layer of the map used in this figure comes from the Thailand—Subnational Administrative Boundaries of the Royal Thai Survey Department. (https://data.humdata.org/dataset/cod-ab-tha). The map was generated by the Quantum GIS: QGIS Software, version 3.24.3.

An age-stratified, population-based, seroepidemiological study was conducted in Chiang Mai, Thailand during March to September 2019. Nine districts were selected as research clusters based on administrative definition, as (1) rural district (*n* = 3): a district with the lowest percentage of urban population, including Wiang Haeng (urban population 0%), Mae On (urban population 0%), and Galyani Vadhana (urban population 0%); (2) urban district (*n* = 3): a district with the highest percentage of urban population, including Mueang (urban population 97%), Saraphi (urban population 98%), and San Sai (urban population 100%); and (3) peri-urban district (*n* = 3): a district with a combination of urban and rural populations, including Hang Dong (urban population 48%), Hot (urban population 51%), and Phrao (urban population 53%), according to the 2010 population and housing census of Thailand (Fig 1) [26].

### Study population

Eligible participants were Chiang Mai residents for at least a year. Participants who suffered from an acute febrile illness within 7 days of enrollment, had primary or secondary immune deficiency, or were receiving immunosuppressive agents were excluded. Within each research cluster, eligible participants were randomly selected from a list of people living in each district from 3 different age groups: (1) adolescents aged 10–20 years; (2) adults aged 21–50 years; and (3) older adults/elderly aged ≥51 years, by computer randomization program. When more than one participant was selected from a single household, the youngest individual was enrolled. If an indicated participant declined to participate in the study, the household was skipped and the next randomly selected participant in the list was approached by recruitment staff. Since only participants aged ≥10 years were enrolled in this study, all previously vaccinated participants had received MBDV during childhood.

### Sample size calculation

The sample size was calculated based on the expected seroprotection of JEV, estimated to be 65% for adolescents, 50% for adults, and 50% for older adults/elderly, accounting for the routine MBDV immunization during childhood, the waning of vaccine-induced immunity, and the chance of exposure to wild JEV of participants in each age group. With a 90% confidence, a 5% margin of error, and a 10% incomplete data, a total of 873 participants were required, including 279 adolescents (*n* = 31 per cluster), 297 adults (*n* = 33 per cluster), and 297 older adults/elderly (*n* = 33 per cluster).

### Data collection

Information, including socio-demographic characteristics, patient-reported history of symptomatic flavivirus infections, including JEV, dengue virus, and zika virus, and history of immunization against JEV, specifically MBDV (JE-VAX, TGPO, Beijing strain) from vaccine booklet or self-reporting were collected during the study visit.

### Sample collection and plaque reduction neutralization test against JEV

Blood samples (5 ml) were collected via venipuncture. Sera were extracted and stored at -20°C until transportation to the Center for Vaccine Development, Institute of Molecular Biosciences, Mahidol University (Bangkok, Thailand) for PRNT to quantify the titer of neutralizing antibody for JEV. Neutralizing antibodies were measured by PRNT_50_ using an established laboratory guideline [27]. To explore the possibility of cross-reaction with dengue virus, neutralizing antibodies using the more specific PRNT_90_ threshold were also calculated. Wild-type JEV Beijing strain were used as an input virus, and LLC-MK2 cells were used to determine PRNT_50_ and PRNT_90_. Briefly, sera were heat-inactivated by incubation at 56°C for 30 min, serial diluted (4-fold), mixed with an equal volume of JEV (Beijing strain), and were inoculated onto triplicate 6-well plates of confluent LLC-MK2 cells. Plaques were counted after incubation for 7 days at 37°C with 5% CO_2_ atmosphere. The final end point neutralization titers were the inverse of the highest serial dilution of serum that can neutralize ≥50% and ≥90% of input JEV (Beijing strain). The titer levels of PRNT_50_ and PRNT_90_ of ≥10 (1/dil) indicated the presence of JEV neutralizing antibody.

### Definition of seroprotection against JEV

The study outcome was an age-stratified proportion of the population, including adolescents, adults and older adults/elderly, seroprotected against JEV. To account for the cross-reactivity of JEV and other flaviviruses, particularly dengue virus in our study setting, we defined JEV seroprotection in this study differently for each of two age groups (10–28 years and >28 years), corresponding to a younger cohort likely to have received MBDV vaccine (JE-VAX, TGPO, Beijing strain) which was introduced into Thailand Expanded Program on Immunization [EPI] in 1990). According to this classification and the PRNT_50_ and PRNT_90_ results, participants were therefore considered JEV seroprotected or not seroprotected, as described in Table 1.

**Table 1:** Protocol-defined seroprotection definitions against Japanese encephalitis virus among study participants.

Age	History of immunization against JEV	PRNT_50_ level^a^	PRNT_90_ level^a^	Interpretation
Aged 10–28 years	Probably received MBDV (Beijing strain) according to Thailand Expanded Program on Immunization	Positive	Positive	• Possibly having previous natural JEV infection and/or persistent immunity from previous MBDV • Interpretation: **JEV seroprotected**
		Positive	Negative	• Never infected with JEV • Possible cross-reactivity from other flaviviruses and/or persistent immunity from previous MBDV • Interpretation: **JEV seroprotected if confirmed MBDV receipt**
		Negative	Negative	• Never infected with JEV • No residual immunity from MBDV • Interpretation: **not JEV seroprotected**
Aged >28 years	Never received MBDV (Beijing strain) according to Thailand Expanded Program on Immunization	Positive	Positive	• Having natural JEV infection • Interpretation: **JEV seroprotected**
		Positive	Negative	• Never infected with JEV • Possible cross-reactivity from other flaviviruses • Interpretation: **not JEV seroprotected**
		Negative	Negative	• Never infected with JEV • Interpretation: **not JEV seroprotected**

Abbreviations: JEV, Japanese encephalitis virus; MBDV, mouse brain-derived Japanese encephalitis virus vaccine; PRNT_50_, 50% plaque reduction neutralization test; PRNT_90_, 90% plaque reduction neutralization test.

^a^The titer levels of PRNT_50_ and PRNT_90_ of ≥10 (1/dil) were considered the presence of neutralizing antibodies to Japanese encephalitis virus.

### Statistical analysis

The age-stratified, protocol-defined, cluster-adjusted proportion (%) and 95% CI of participants with JEV seroprotection were calculated. We also performed an estimation for JEV seroprevalence based on PRNT_50_ ≥10 (1/dil) and PRNT_90_ ≥10 (1/dil) definitions. Univariable generalized estimating equation (GEE) population-average model with an exchangeable correlation structure was performed to determine the socio-demographic and immunization history risk factors associated with JEV seroprotection for participants, adjusted for the cluster (district) effect, in each age group separately. The modelling approach therefore considered both differences between individuals within clusters and between clusters in the final estimates and their standard errors [28]. Covariates demonstrating a *P* <0.20 were included in a multivariable model. In addition, supplementary analyses to identify the associated factors of JEV seroprotection based on PRNT_50_ and PRNT_90_ definitions were conducted with similar processes. A two-tailed *P* <0.05 was considered to be statistically significant. All statistical analyses were performed using Stata statistical software, version 17.0 (StataCorp LP, College Station, TX, USA).

## Results

### Characteristic of study participants

During the study period, a total of 873 participants, including 279 adolescents, 297 adults, and 297 older adults/elderly, were enrolled from nine research clusters (S1 Fig). The characteristics of study participants are summarized in Table 2.

**Table 2:** Characteristics of study participants.

Characteristics^a^	Adolescents (*n* = 279)	Adults (*n* = 297)	Older adults/elderly (*n* = 297)
***Socio-demographic characteristics***			
Age, years	14.7 (12.5–17.3)	36.6 (27.8–45.2)	61.4 (56.3–66.6)
Male sex	161 (57.7)	130 (43.8)	110 (37.0)
Home address			
Rural districts	93 (33.3)	99 (33.3)	99 (33.3)
Urban districts	93 (33.3)	99 (33.3)	99 (33.3)
Peri-urban districts	93 (33.3)	99 (33.3)	99 (33.3)
Household income (*n* = 870)	(*n* = 276)	(*n* = 297)	(*n* = 297)
< 500 USD/month	164 (59.4)	154 (51.8)	217 (73.1)
≥ 500 USD/month	112 (40.6)	143 (48.2)	80 (26.9)
Number of household member			
1–2	13 (4.6)	76 (25.6)	119 (40.1)
3–5	205 (73.5)	187 (63.0)	134 (45.1)
>5	61 (21.9)	34 (11.4)	44 (14.8)
Ever had dengue virus infection^b^	22 (7.9)	32 (10.8)	21 (7.1)
***History of immunization against JEV***			
Ever received MBDV^c^			
Yes	195 (69.9)	2 (0.7)	0 (0)
No	18 (6.4)	216 (72.7)	297 (100)
Not sure	66 (23.7)	79 (26.6)	0 (0)
Duration from last dose of MBDV to enrollment^c^, years	11.0 (9.5–13.2)	24.8 (23.5–26.0)	NA
Number of MBDV received^d^ (*n* = 197)	(*n* = 195)	(*n* = 2)	NA
2 doses	4 (2.1)	1 (50.0)	
3 doses	183 (93.8)	1 (50.0)	
4 doses	8 (4.1)	0 (0)	

Abbreviations: JEV, Japanese encephalitis virus; MBDV, mouse brain-derived JEV vaccine; NA, not applicable; USD, US dollar.

^a^Data were presented as n (%) for categorical variables, and median (interquartile range) for continuous variables.

^b^From patient-reported history.

^c^From vaccine booklet reviewing or patient-reported history.

^d^Among all participants received mouse brain-derived JEV vaccine.

For adolescents, 58% were male, and the median age was 15 (interquartile range [IQR]: 13–17) years. By patient-reported history, 22 adolescents (8%) had previous dengue virus infection, of whom 20 (91%) had laboratory-confirmed diagnosis, and 16 (73%) were admitted to the hospital. None reported previous history of symptomatic JEV or zika virus infection. There were 195 adolescents (70%) who had received MBDV, with a median duration from the last dose of vaccine to enrollment of 11 (IQR: 10–13) years, by vaccine booklet review or patient-reported history (Table 2).

Among adults, 44% were male, and the median age was 37 (IQR: 28–45) years. By patient-reported history, 32 (11%) had dengue virus infection history, of whom 28 (88%) had laboratory-confirmed diagnosis, and 20 (63%) were admitted to the hospital. One adult reported previous infection with zika virus, and none with symptomatic JEV infection. Two (1%) had received MBDV, with a duration from the last dose of vaccine to enrollment of 24 and 26 years (Table 2).

For older adults/elderly, 37% were male, and the median age was 61 (IQR: 56–67) years. Twenty-one (7%) had reported history of dengue virus infection, of whom 17 (81%) had laboratory-confirmed diagnosis, and 11 (52%) were admitted to the hospital. None reported previous history of symptomatic JEV or zika virus infection. There were no participants in this age group ever received MBDV according to Thailand EPI (Table 2).

### Age-stratified seroepidemiology of JEV

**Fig 2:**
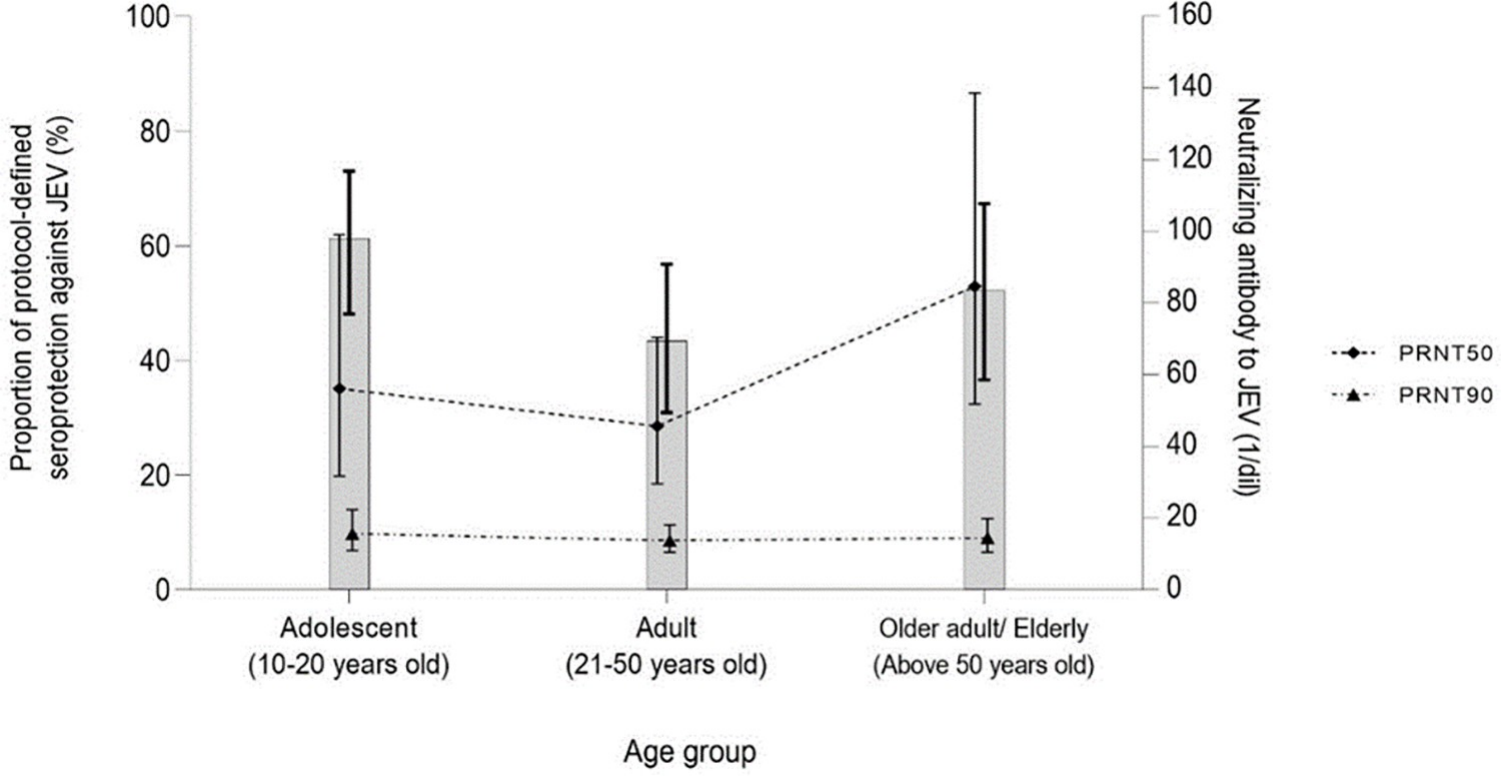
Age-stratified seroepidemiology of Japanese encephalitis virus among study participants. Abbreviations: JEV, Japanese encephalitis virus; PRNT_50_, 50% plaque reduction neutralization test; PRNT_90_, 90% plaque reduction neutralization test. Bar chart represents the proportion of study participants with protocol-defined seroprotection against JEV. Line chart represents the neutralizing antibody to JEV based on PRNT_50_ (dashed line) and PRNT_90_ (dotted and dashed line). Vertical line represents the 95% confidence interval of each parameter.

Based on the protocol definition, 171/279 adolescents (61%; 95% CI: 48–73%), 129/297 adults (43%; 95% CI: 31–57%), and 155/297 (52%; 95% CI: 37–67%) demonstrated seroprotection to JEV. The summary of seroepidemiology of JEV, according to the PRNT_50_ and PRNT_90_ definitions, are demonstrated in Table 3. The GMT of neutralizing antibodies to JEV using PRNT_50_ were 56.1 (95% CI: 31.7–99.1), 45.6 (95% CI: 29.5–70.4), and 84.7 (95% CI: 51.8–138.6) (1/dil); and PRNT_90_ were 15.6 (95% CI: 10.9–22.3), 13.7 (95% CI:10.4–18.1), 14.4 (95% CI:10.4–19.9) (1/dil) among adolescents, adults, and older adults/elderly, respectively (Fig 2).

**Table 3:** Summary of age-stratified seroepidemiology of Japanese encephalitis virus, based on all three serological definitions against Japanese encephalitis virus.

Parameter^a^	Protocol definition	PRNT_50_ definition	PRNT_90_ definition
***Proportion of JEV seroprotection***			
Adolescents (*n* = 279)	171 (61.3)	188 (67.4)	122 (43.7)
Adults (*n* = 297)	129 (43.4)	201 (67.7)	127 (42.8)
Older adults/elderly (*n* = 297)	155 (52.2)	260 (87.5)	155 (52.2)
***Geometric mean titer*, *(1/dil)***			
Adolescents (*n* = 279)	NA	56.1 (31.7–99.1)	15.6 (10.9–22.3)
Adults (*n* = 297)	NA	45.6 (29.5–70.4)	13.7 (10.4–18.1)
Older adults/elderly (*n* = 297)	NA	84.7 (51.8–138.6)	14.4 (10.4–19.9)

Abbreviations: JEV, Japanese encephalitis virus; NA, not applicable; PRNT_50_, 50% plaque reduction neutralization test; PRNT_90_, 90% plaque reduction neutralization test.

^a^Data were presented as n (%) for categorical variables, and geometric mean titer (95% confidence interval) for continuous variables.

### Associated factors of seroprotection against JEV

In the multivariable GEE population-averaged model for adolescents, living in peri-urban districts, having history of prior dengue virus infection, and previously receiving at least one dose of MBDV were significantly associated with JEV seropositivity (Table 4). Focusing on adults, older age and male sex demonstrated a significant association with JEV seroprotection (Table 4). For older adults/elderly, male sex was the only significant associated factor (Table 4). Analyses to identify factors associated with JEV seropositivity based on PRNT_50_ and PRNT_90_ definitions are shown in the S1 and S2 Tables, respectively.

**Table 4:** Associated factors of protocol-defined seropositivity against Japanese encephalitis virus among study participants, stratified by age group.

Characteristics	Adolescents (*n* = 279)				Adults (*n* = 297)				Older adults / elderly (*n* = 297)			
	Univariable^a^		Multivariable^a^		Univariable^a^		Multivariable^a^		Univariable^a^		Multivariable^a^	
	Crude OR (95% CI)	*P*	aOR (95% CI)	*P*	Crude OR (95% CI)	*P*	aOR (95% CI)	*P*	Crude OR (95% CI)	*P*	aOR (95% CI)	*P*
Age, per one year increased	0.93 (0.86–1.02)	0.12	0.97 (0.88–1.08)	0.63	1.03 (1.01–1.05)	0.003	1.03 (1.01–1.05)	0.002	1.03 (1.01–1.06)	0.03	1.03 (1.00–1.06)	0.07
Male sex (*vs*. female sex)	0.70 (0.36–1.37)	0.30			2.08 (1.68–2.57)	<0.001	2.13 (1.71–2.66)	<0.001	1.62 (1.08–2.41)	0.02	1.63 (1.06–2.50)	0.03
Home address												
Urban districts	Ref		Ref		Ref		Ref		Ref			
Rural districts	1.85 (0.87–3.90)	0.11	1.60 (0.82–3.11)	0.17	1.19 (0.48–2.93)	0.70	0.75 (0.32–1.75)	0.50	0.85 (0.18–4.08)	0.84		
Peri-urban districts	3.20 (1.41–7.28)	0.005	2.76 (1.37–5.57)	0.004	2.59 (1.21–5.54)	0.01	1.93 (0.84–4.45)	0.12	1.00 (0.42–2.36)	1.00		
Household income < 500 USD/month (*vs*. ≥500 USD/month)	1.76 (0.99–3.11)	0.05	2.10 (0.93–4.76)	0.07	2.10 (1.09–4.05)	0.03	1.80 (0.88–3.67)	0.11	1.29 (0.89–1.88)	0.18	1.22 (0.82–1.81)	0.32
Number of household member < 3 people (*vs*. ≥3 people)	1.44 (0.57–3.68)	0.44			1.59 (1.21–2.08)	0.001	1.05 (0.70–1.57)	0.82	0.96 (0.73–1.26)	0.77		
Ever (*vs*. never) had dengue virus infection^b^	2.41 (0.98–5.94)	0.06	3.01 (1.08–8.39)	0.04	1.19 (0.67–2.10)	0.55			0.83 (0.34–2.01)	0.68		
Ever (*vs*. never) received MBDV vaccine^c^	1.90 (1.12–3.22)	0.02	2.27 (1.24–4.17)	0.008								

Abbreviations: aOR, adjusted odds ratio; MBDV, mouse brain-derived JEV vaccine; OR, odds ratio; Ref, reference group; USD, US dollar; 95% CI, 95% confidence interval.

^a^Univariable generalized estimating equation (GEE) population-averaged model was performed to determine the socio-demographic and immunization history risk factors associated with JEV seroprotection, adjusted for the effects of clustering, for participants in each age group separately. Covariates demonstrating a *P* <0.20 were included in a multivariable model. Covariates included in the final model are as listed in the table.

^b^From patient-reported history.

^c^From vaccine booklet reviewing or patient-reported history.

## Discussion

This study demonstrates that 61%, 43%, and 52% of general adolescents, adults, and older adults/elderly living Chiang Mai, Thailand–a highly endemic area for JE–demonstrated seroprotection to JEV based on a definition incorporating vaccination history and neutralizing antibody concentrations circulating in the blood of study participants. Living in peri-urban districts, having prior dengue virus infection, and previously receiving at least one dose of MBDV were associated with JEV seroprotection in adolescents, whereas older age and male sex were the associated factors among adults; and only male sex for older adults/elderly. Our results suggest that, despite the commendable vaccination effort over the past 28 years, approximately half of general population living in hyperendemic areas may remain susceptible to JEV infection.

The variation of age-stratified seroprotection to JEV across 3 groups of population demonstrated in this study was similar to that observed in other JE high-endemic countries [29,30]. In Japan, a seroprevalence study surveying JEV neutralizing antibodies among general populations in the National Epidemiological Surveillance of Vaccine Preventable Diseases (2004) showed that JEV seroprotection (PRNT_50_ ≥10 [1/dil]) was highest among adolescents aged 10–19 years with a seroprevalence of >75%, which gradually declined to the lowest proportion of <25% among adults aged 40–49 years, and then increased to peak in older adults/elderly aged 60–69 years with a seroprevalance of >75% [29]. Likewise, a nationwide population-based study in Taiwan investigated the age-specific seroprevalence of JEV neutralizing antibodies among general populations from the National Health Interview Survey (2002) and found that the seropositivity against JEV (PRNT_50_ ≥10 [1/dil]) peaked in adolescents aged 16–21 years with a seroprevalence of 74%, which declined to a minimum of 54% in adults aged 33–39 years, and then rebounded to the highest proportion of 86% in older adults/elderly aged ≥50 years [30]. These “U-shaped” patterns might be attributable to a childhood JE immunization among adolescents, and history of natural infection in older adults/elderly, leading to a high JEV seroprevalence in these groups. Low seroprevalence in the adult group could be due to a waning of JE vaccine-induced neutralizing antibodies, incomplete immunization during childhood, and lack of exposure to wild JEV due to reduced force of infection in the population as a whole following broader societal change [29,30].

In contrast, a Korean study conducted in adults and older adults/elderly aged 30–69 years in 2010 showed that the seroprevalence against JEV was very high, with an average of 98%, among study participants of all age groups [31]. The high seropositivity noted in the Korean study might be because of a long-standing immunization with an inactivated mouse brain-derived JEV vaccine (Nakayama strain) in the National Immunization Program of South Korea for all children annually since the 1980s, and a high incidence of natural JEV infections in the country [31,32]. In addition, different JEV vaccine strain (Beijing *vs*. Nakayama) of MBDV between our country and South Korea, environmental factors including residences, sanitary conditions, occupations, as well as geographic risk of JE transmission, and geographic variation in vaccine coverage could yield the differences in the dynamic pattern of JEV seroprevalence between this Korean study results and ours [31,32].

In Thailand, Japan, and Taiwan, the incidence of JE has significantly declined after the introduction of JE vaccine into the National Immunization Program. However, there are still a report of laboratory-confirmed JE cases in these countries every year [1]. Notably, the age distribution of JE cases shifted from mainly children to adults [1], which corresponded to the results of JEV seroprevalence survey in this study as well as in the Japan and Taiwan studies that the proportion of population with JEV seroprotection was lowest among adults [29,30]. A booster or catch-up dose of live-attenuated or inactivated JEV vaccine in this group of the population might be considered to reduce the incidence of disease.

The cross-reactivity of immunoglobulin G antibodies across members of the flavivirus family has been well documented. This causes a challenge to assess seroprevalence of each flavivirus in the areas where multiple virus members co-circulate, particularly JEV and dengue virus [20,21]. A previous study conducted in several countries in Southeast Asia, including Indonesia, Malaysia, Philippines and Vietnam, noted that JEV seroprevalence estimates (PRNT_50_ ≥10 [1/dil]) was significant higher in children who had dengue infection in the past, compared with those had never experienced a dengue infection. This finding is a consequence of the cross-neutralization of JEV and dengue virus assays [22]. Thus, PRNT_90_, a more stringent threshold which was used in this study, may be preferred for seroepidemiological studies in the areas that have high levels of JEV and dengue virus endemicity, and have a high JEV vaccination coverage [24,25], such as Thailand.

We identified factors associated with JEV seroprotection which varied by age group. Among adolescents, living in peri-urban districts were positively associated with JEV seropositivity. Peri-urban environments may provide increased opportunity of acquiring natural JEV infection through proximity with vertebrate host animals. Indeed, in the past, JE was considered primarily a rural disease. However, as a result of peri-urban growth, change in human activities, change of agricultural practices, animal vectors (e.g., mosquitoes) and amplifying hosts (e.g., birds, pigs), as well as change of climate, the distribution of JEV continues to evolve [33]. The shift of JEV infections from rural to peri-urban areas has also been documented in countries including South Korea, Taiwan, China, and Singapore [33,34]. The association between JEV seropositivity and a history of prior dengue virus infection reflects the co-circulation of multiple flaviviruses, particularly JEV and dengue virus, in our setting. We also found that JEV seroprotection increased with age among adults, the majority of whom had never been immunized with MBDV, suggesting that natural JEV infection had resulted in durable immunological responses [29,30]. Furthermore, since most of Thai men are agricultural workers working in farms and rice fields—the common breeding ground of *Culex* mosquitoes, we also noted the association between male sex and JEV seroprotection among adults and older adults/elderly in this study [3].

This study has some strengths. We investigated the seroepidemiology of JEV in a large number of residents living in a highly endemic area for JE. Additionally, we recruited our study participants from three different geographic locations (rural, urban, and peri-urban), and three different age groups (adolescents, adults, and older adults/elderly) to effectively represent the seroepidemiology of JEV in these populations. We used a computer randomization program to minimize selection bias. Importantly, we used a stringent definition, accounting for age, previous MBDV immunization, as well as the PRNT_50_ and PRNT_90_ results, to define JEV seroprotection to diminish the possibility of cross-reactivity of neutralizing antibodies from other flaviviruses, particularly dengue virus, which are also endemic in Thailand.

Nevertheless, this study still contains some limitations. Firstly, since there are currently no laboratory assays to differentiate between vaccine-induced and natural infection-induced neutralizing antibodies against JEV, we were unable to make a definite conclusion regarding the causes of acquisition of JEV immunity, particularly for adolescents and young adults who had been vaccinated but also had potential to acquire natural JEV infection. Despite recruiting study participants from different clusters, our convenience sample did not recruit in a random, representative manner, and therefore seroprevalence in other areas of Thailand may differ. In addition, we did not apply a design effect in the sample size calculation due to lack of information on inter-cluster variability. There is a possibility of recall bias as the information on childhood vaccination and history of flavivirus infections relied primarily on self-reports. Also, participants with asymptomatic or mild infection might not recognize their illness, and this could significantly underestimate the prevalence of previous flavivirus infections, particularly JEV, in our study population. Information which might influence JEV seroepidemiology, such as travel or relocation history, were not collected. Finally, since we conducted this study in only Chiang Mai province, our results and interpretations might not be generalizable nationwide for which a nationally representative study would be needed.

In summary, we consider approximately half of general population of Chiang Mai, Thailand, exhibited serological profiles protective from natural JEV infection. Ongoing nationwide surveillance on the seroepidemiology of JEV is an important strategy to understand the evolving population-level immunity to JEV, to guide the implementation of JE control measures, and to help formulating the appropriate recommendations on JE immunization for our country in the near future.

## Supporting information

S1 Table Associated factors of seropositivity against Japanese encephalitis virus among study participants based on PRNT_50_ definition, stratified by age group. (DOCX)

S2 Table Associated factors of seropositivity against Japanese encephalitis virus among study participants based on PRNT_90_ definition, stratified by age group. (DOCX)

S1 Fig Flow diagram of study participants. (DOCX)

S1 File Dataset for the study. (XLS)
