# Supplementary material for: Seroepidemiological study of Japanese encephalitis virus in Chiang Mai: Immunity and susceptibility 28 years after introduction of a vaccination programme
Source: PLoS Negl Trop Dis. 2022 Aug 1;16(8):e0010674. doi: 10.1371/journal.pntd.0010674 (PMC9371339; doi:10.1371/journal.pntd.0010674)
Supplement: S1 Table — (DOCX) [file pntd.0010674.s001.docx]

**Supplementary Table 1. Associated factors of seropositivity against Japanese encephalitis virus among study participants based on PRNT_50_ definition, stratified by age group**

| **Characteristics** | **Adolescents (*n* = 279)** | | | |  | **Adults (*n* = 297)** | | | |  | **Older adults / elderly (*n* = 297)** | | | |
| --- | --- | --- | --- | --- | --- | --- | --- | --- | --- | --- | --- | --- | --- | --- |
|  | **Univariable**^a^ | | **Multivariable**^a^ | |  | **Univariable**^a^ | | **Multivariable**^a^ | |  | **Univariable**^a^ | | **Multivariable**^a^ | |
|  | **Crude OR**  **(95% CI)** | ***P*** | **aOR**  **(95% CI)** | ***P*** |  | **Crude OR**  **(95% CI)** | ***P*** | **aOR**  **(95% CI)** | ***P*** |  | **Crude OR**  **(95% CI)** | ***P*** | **aOR**  **(95% CI)** | ***P*** |
| Age, per one year increased | 0.98  (0.91-1.06) | 0.61 |  |  |  | 1.05  (1.02-1.09) | 0.003 | 1.07  (1.03-1.10) | <0.001 |  | 1.04  (0.99-1.10) | 0.14 | 1.03  (1.01-1.07) | 0.02 |
| Male sex  (*vs.* female sex) | 0.65  (0.33-1.30) | 0.23 |  |  |  | 1.67  (1.01-2.74) | 0.04 | 1.87  (1.12-3.11) | 0.02 |  | 1.27  (0.65-2.48) | 0.49 |  |  |
| Home address |  |  |  |  |  |  |  |  |  |  |  |  |  |  |
| Urban districts | Ref |  | Ref |  |  | Ref |  | Ref |  |  | Ref |  | Ref |  |
| Rural districts | 1.91  (1.06-3.44) | 0.03 | 1.65  (0.83-3.30) | 0.15 |  | 1.13  (0.39-3.28) | 0.82 | 0.73  (0.33-1.63) | 0.45 |  | 0.23  (0.04-1.34) | 0.10 | 0.12  (0.01-0.90) | 0.04 |
| Peri-urban districts | 2.82  (1.19-6.72) | 0.02 | 2.45  (1.06-5.67) | 0.04 |  | 3.41  (1.21-9.59) | 0.02 | 2.87  (1.24-6.61) | 0.01 |  | 4.26  (1.74-10.45) | 0.002 | 1.48  (0.32-6.80) | 0.61 |
| Household income  < 500 USD/month (*vs.* ≥500 USD/month) | 1.96  (0.99-3.89) | 0.06 | 1.95  (0.80-4.75) | 0.14 |  | 2.02  (0.94-4.33) | 0.07 | 1.68  (0.80-3.50) | 0.17 |  | 2.87  (1.37-6.00) | 0.005 | 3.91  (2.07-7.39) | <0.001 |
| Number of household member < 3 people (*vs*. ≥3 people) | 1.16  (0.44-3.00) | 0.77 |  |  |  | 1.78  (1.27-2.48) | 0.001 | 1.19  (0.74-1.93) | 0.47 |  | 0.95  (0.51-1.78) | 0.88 |  |  |
| Ever (*vs.* never) had dengue virus infection^b^ | 3.55  (0.68-18.50) | 0.13 | 4.07  (0.83-19.98) | 0.09 |  | 1.83  (0.83-4.04) | 0.13 | 2.64  (1.11-6.29) | 0.03 |  | 0.61  (0.25-1.46) | 0.27 |  |  |
| Ever (*vs.* never) received MBDV vaccine^c^ | 0.82  (0.49-1.38) | 0.46 |  |  |  |  |  |  |  |  |  |  |  |  |

Abbreviations: aOR, adjusted odds ratio; MBDV, mouse brain-derived JEV vaccine; OR, odds ratio; Ref, reference group; USD, US dollar; 95% CI, 95% confidence interval.

^a^Univariable generalized estimating equation (GEE) population-averaged model was performed to determine the socio-demographic and immunization history risk factors associated with JEV seroprotection, adjusted for the effects of clustering, for participants in each age group separately. Covariates demonstrating a *P* <0.20 were included in a multivariable model. Covariates included in the final model are as listed in the table.

^b^From patient-reported history.

^c^From vaccine booklet reviewing or patient-reported history.
