# Supplementary material for: Seroepidemiological study of Japanese encephalitis virus in Chiang Mai: Immunity and susceptibility 28 years after introduction of a vaccination programme
Source: PLoS Negl Trop Dis. 2022 Aug 1;16(8):e0010674. doi: 10.1371/journal.pntd.0010674 (PMC9371339; doi:10.1371/journal.pntd.0010674)
Supplement: S2 Table — (DOCX) [file pntd.0010674.s002.docx]

**Supplementary Table 2. Associated factors of seropositivity against Japanese encephalitis virus among study participants based on PRNT_90_ definition, stratified by age group**

| **Characteristics** | **Adolescents (*n* = 279)** | | | |  | **Adults (*n* = 297)** | | | |  | **Older adults / elderly (*n* = 297)** | | | | |
| --- | --- | --- | --- | --- | --- | --- | --- | --- | --- | --- | --- | --- | --- | --- | --- |
|  | **Univariable**^a^ | | **Multivariable**^a^ | |  | **Univariable**^a^ | | **Multivariable**^a^ | |  | **Univariable**^a^ | | | **Multivariable**^a^ | |
|  | **Crude OR**  **(95% CI)** | ***P*** | **aOR**  **(95% CI)** | ***P*** |  | **Crude OR**  **(95% CI)** | ***P*** | **aOR**  **(95% CI)** | ***P*** |  | **Crude OR**  **(95% CI)** | ***P*** | **aOR**  **(95% CI)** | | ***P*** |
| Age, per one year increased | 1.03  (0.93-1.13) | 0.59 |  |  |  | 1.03  (1.01-1.05) | 0.002 | 1.04  (1.02-1.06) | <0.001 |  | 1.03  (1.00-1.06) | 0.03 | 1.03  (1.00-1.06) | | 0.08 |
| Male sex  (*vs.* female sex) | 0.73  (0.37-1.41) | 0.35 |  |  |  | 2.06  (1.64-2.60) | <0.001 | 2.17  (1.72-2.74) | <0.001 |  | 1.62  (1.08-2.41) | 0.02 | 1.63  (1.06-2.50) | | 0.03 |
| Home address |  |  |  |  |  |  |  |  |  |  |  |  |  | |  |
| Urban districts | Ref |  | Ref |  |  | Ref |  | Ref |  |  | Ref |  |  | |  |
| Rural districts | 1.20  (0.64-2.25) | 0.57 | 0.93  (0.45-1.96) | 0.86 |  | 1.19  (0.47-3.04) | 0.71 | 0.72  (0.29-1.78) | 0.48 |  | 0.85  (0.18-4.08) | 0.84 |  | |  |
| Peri-urban districts | 2.31  (1.24-4.28) | 0.008 | 2.09  (1.11-3.94) | 0.02 |  | 2.71  (1.27-5.79) | 0.01 | 2.03  (0.90-4.57) | 0.09 |  | 1.00  (0.42-2.36) | 1.00 |  | |  |
| Household income  < 500 USD/month (*vs.* ≥500 USD/month) | 1.54  (0.86-2.74) | 0.14 | 1.57  (0.77-3.19) | 0.22 |  | 2.08  (1.08-4.00) | 0.03 | 1.78  (0.87-3.61) | 0.11 |  | 1.29  (0.89-1.88) | 0.18 | 1.22  (0.82-1.81) | | 0.32 |
| Number of household member < 3 people (*vs*. ≥3 people) | 1.35  (0.49-3.73) | 0.56 |  |  |  | 1.53  (1.19-1.97) | 0.001 | 0.99  (0.66-1.48) | 0.97 |  | 0.96  (0.73-1.26) | 0.77 |  | |  |
| Ever (*vs.* never) had dengue virus infection^b^ | 1.57  (0.64-3.85) | 0.33 |  |  |  | 1.22  (0.69-2.14) | 0.50 |  |  |  | 0.83  (0.34-2.01) | 0.68 |  | |  |
| Ever (*vs.* never) received MBDV vaccine^c^ | 0.66  (0.46-0.96) | 0.03 | 0.73  (0.50-1.05) | 0.09 |  |  |  |  |  |  |  |  |  | |  |

Abbreviations: aOR, adjusted odds ratio; MBDV, mouse brain-derived JEV vaccine; OR, odds ratio; Ref, reference group; USD, US dollar; 95% CI, 95% confidence interval.

^a^Univariable generalized estimating equation (GEE) population-averaged model was performed to determine the socio-demographic and immunization history risk factors associated with JEV seroprotection, adjusted for the effects of clustering, for participants in each age group separately. Covariates demonstrating a *P* <0.20 were included in a multivariable model. Covariates included in the final model are as listed in the table.

^b^From patient-reported history.

^c^From vaccine booklet reviewing or patient-reported history.
