# Supplementary figures and images for: Seroepidemiological study of Japanese encephalitis virus in Chiang Mai: Immunity and susceptibility 28 years after introduction of a vaccination programme
Source: PLoS Negl Trop Dis. 2022 Aug 1;16(8):e0010674. doi: 10.1371/journal.pntd.0010674 (PMC9371339; doi:10.1371/journal.pntd.0010674)

**Supplementary Figure 1.** Flow diagram of study participants.


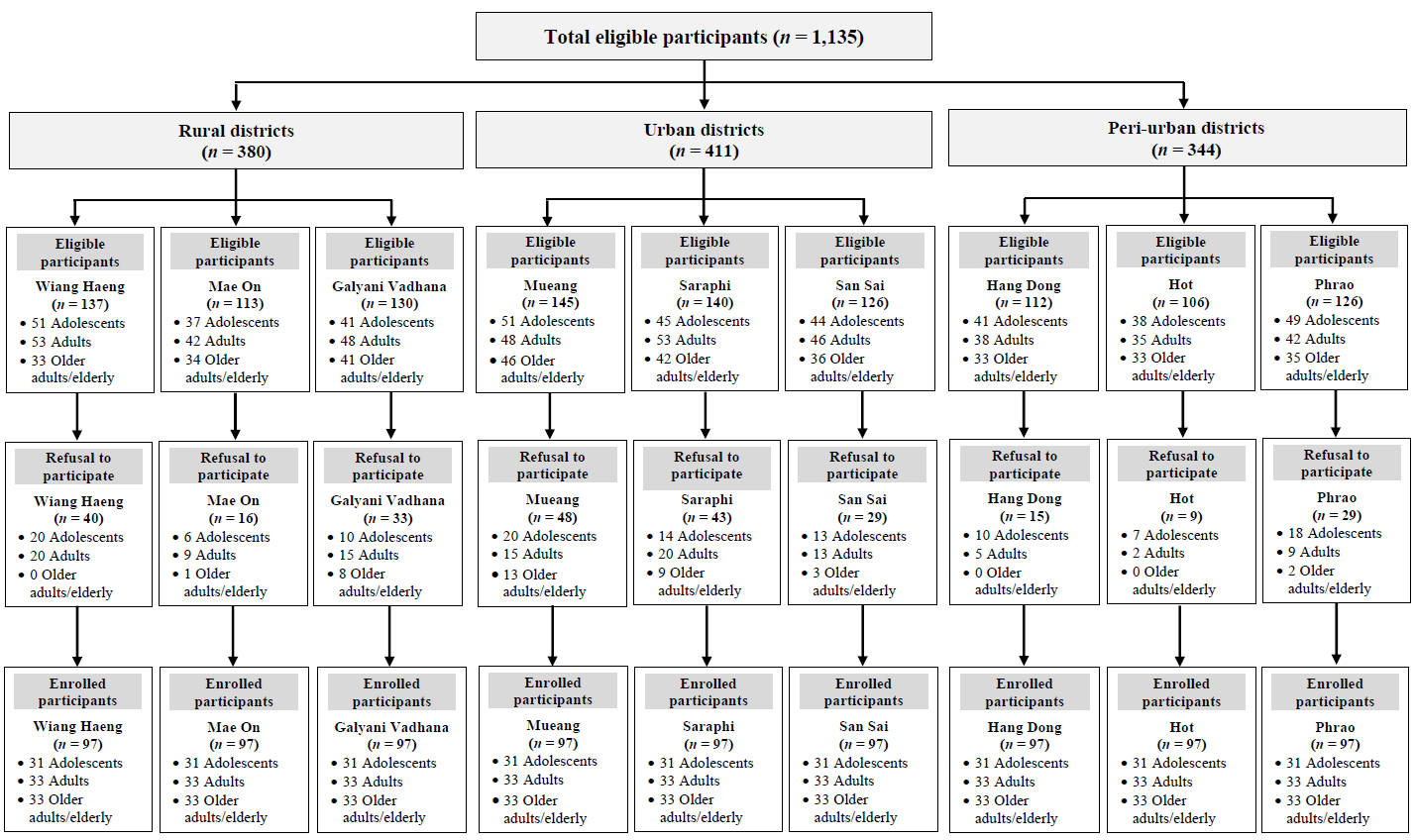

Supplement: S1 Fig — (DOCX) [file pntd.0010674.s003.docx]
